# Updated annotation of the wild strawberry *Fragaria vesca* V4 genome

**DOI:** 10.1038/s41438-019-0142-6

**Published:** 2019-05-01

**Authors:** Yongping Li, Mengting Pi, Qi Gao, Zhongchi Liu, Chunying Kang

**Affiliations:** 1 Key Laboratory of Horticultural Plant Biology (Ministry of Education), College of Horticulture and Forestry Sciences, Huazhong Agricultural University, Wuhan, Hubei, 430070 China; 2 Department of Cell Biology and Molecular Genetics, University of Maryland, College Park, MD 20742 USA

**Keywords:** Transcriptomics, Non-model organisms

## Abstract

The diploid strawberry *Fragaria vesca* serves as an ideal model plant for cultivated strawberry (*Fragaria* *×* *ananassa*, 8*x*) and the *Rosaceae* family. The *F. vesca* genome was initially published in 2011 using older technologies. Recently, a new and greatly improved *F. vesca* genome, designated V4, was published. However, the number of annotated genes is remarkably reduced in V4 (28,588 genes) compared to the prior annotations (32,831 to 33,673 genes). Additionally, the annotation of V4 (v4.0.a1) implements a new nomenclature for gene IDs (FvH4_XgXXXXX), rather than the previous nomenclature (geneXXXXX). Hence, further improvement of the V4 genome annotation and assigning gene expression levels under the new gene IDs with existing transcriptome data are necessary to facilitate the utility of this high-quality *F. vesca* genome V4. Here, we built a new and improved annotation, v4.0.a2, for *F. vesca* genome V4. The new annotation has a total of 34,007 gene models with 98.1% complete Benchmarking Universal Single-Copy Orthologs (BUSCOs). In this v4.0.a2 annotation, gene models of 8,342 existing genes are modified, 9,029 new genes are added, and 10,176 genes possess alternatively spliced isoforms with an average of 1.90 transcripts per locus. Transcription factors/regulators and protein kinases are globally identified. Interestingly, the transcription factor family *FAr-red-impaired Response 1* (*FAR1*) contains 82 genes in v4.0.a2 but only two members in v4.0.a1. Additionally, the expression levels of all genes in the new annotation across a total of 46 different tissues and stages are provided. Finally, miRNAs and their targets are reanalyzed and presented. Altogether, this work provides an updated genome annotation of the *F. vesca* V4 genome as well as a comprehensive gene expression atlas with the new gene ID nomenclature, which will greatly facilitate gene functional studies in strawberry and other evolutionarily related plant species.

## Marking up the strawberry genome

An updated annotation of the wild strawberry genome includes over nine thousand new genes. Since the genome sequence of the wild strawberry was first published in 2011, technological improvements have led to various refinements and updates. Chunying Kang and colleagues at Huazhong Agricultural University in Wuhan, China, found that a large number of genes were either absent or inaccurately described in the annotation of the latest wild strawberry genome. They annotated 5,419 more protein-coding genes, including 139 transcription factor and 92 protein kinase encoding genes, and carried out a comprehensive analysis of the expression patterns of all genes in the new annotation. They also identified microRNAs that contribute to regulate gene expression. These data will aid future comparative and functional studies in widely grown hybrid strawberry species.

## Introduction

The cultivated strawberry (*Fragaria* *×* *ananassa*, 8*x*) is an economically important crop worldwide. The diploid strawberry *Fragaria vesca* serves as an ideal model plant for cultivated strawberry as well as the *Rosaceae* family. *F. vesca* is the most widely distributed diploid *Fragaria* species naturally and is considered to be one of the progenitors of the cultivated strawberry^1^. Due to these features, its genome was initially assembled using short reads (<300 bp) of DNA sequence from a fourth-generation inbred line of Hawaii 4 (*F. vesca ssp. vesca*)^2^, called FvH4, and reassembled based on dense linkage maps of the North American diploid *F. vesca ssp. bracteata*^3^, called Fvb. Recently, the *F. vesca* V4 genome, a near-complete genome with a contig N50 of approximately 7.9 million base pairs (Mb), was assembled using long reads generated by Pacific Biosciences (PacBio) from Hawaii 4^4^. This high-quality genome provides a better reference for genomic and transcriptomic analyses of *F. vesca*.

Accurate and complete gene annotation is an important aspect of a good reference genome. To achieve this goal, one genome usually undergoes several rounds of reannotation. For instance, the 11th annotation of the *Arabidopsis thaliana* genome was released last year^5^. Prior to the new V4 genome of *F. vesca*, the old genome assemblies (V1 and V2) underwent four versions of annotations, including v1.1^2^ and v1.1.a2^6^ for FvH4 and v2.0.a1^3^ and v2.0.a2^7^ for Fvb. The numbers of total protein-coding genes in these annotations are 32,831 (v1.1), 33,496 (v1.1.a2), 33,673 (v2.0.a1), and 33,538 (v2.0.a2). In contrast, the new V4 genome, while containing 24.96 Mb of new sequences, possesses only 28,588 genes based on annotation v4.0.a1^4^. In other words, a few thousand genes were missed in v4.0.a1. Thus, genome reannotation of the V4 genome may uncover a great number of novel genes. In addition, the older genomes (V1 and V2) and their first four annotations name genes as geneXXXXX, while the new genome and its v4.0.a1 annotation implement a different gene naming system, FvH4_XgXXXXX, where the X before g is the linkage group number. FvH4_XgXXXXX is a preferable naming system because it indicates the gene location in the chromosome. However, several previous studies reported gene expression levels using gene IDs following the older nomenclature^8–10^. To make use of these valuable data, it is highly desirable to establish a digital gene expression atlas using the new gene IDs.

Previously, we created a high-quality annotation, v2.0.a2, for the Fvb genome using a sophisticated annotation pipeline^7^. In this study, we mapped this annotation to the V4 genome and reran the pipeline with the previous datasets, including a total of 97 RNA-seq libraries generated from floral and fruit tissues at different developmental stages, as well as from seedlings, leaves, meristems, and roots^7–9,11,12^. Combining these two types of results together, an updated annotation, v4.0.a2, including 34,007 protein-coding genes with 98.1% complete Benchmarking Universal Single-Copy Orthologs (BUSCOs) was obtained. Then, the newly added genes were carefully characterized. Additionally, the expression levels of all the genes across different floral and fruit tissue types are provided in the supplementary table. Moreover, a total of 84 known and 63 novel miRNAs were identified, and their targets were predicted. Overall, the new annotation and gene expression data provide valuable data resources for future studies.

## Results and discussion

### Reannotation of the *F. vesca* V4 genome using our existing pipeline

Continuous refinement and routine updates of annotation are essential for functional genome research. Previously, we created an updated annotation of the Fvb genome, called v2.0.a2, which is of high quality with 95.7% complete BUSCOs^7^. To reannotate the *F. vesca* V4 genome, the v2.0.a2 annotation was first aligned to the V4 genome using the FLO pipeline (https://github.com/wurmlab/flo) with the default parameters. Consequently, a total of 33,272 genes were aligned to the V4 genome, accounting for 99.2% of the v2.0.a2 genes. Because the V4 genome contains ~25 Mb of sequences that are newly augmented^4^, we reran the annotation pipeline using the available transcriptome datasets. We used a total of 97 RNA-seq libraries generated from 46 different tissue types in *F. vesca*^7–9,11,12^, including tissues from fruit and flowers at different developmental stages, as well as from seedlings, leaves, meristems, and roots. Among these datasets, SAM (shoot apical meristem), FM (flower meristem), REM (receptacle meristem), Root, and Root_P (roots challenged with the pathogen *Phytophthora cactorum*) have three biological replicates, while the rest of the tissues have two biological replicates. In total, there are 2.9 billion RNA-seq short reads and 82,360 full-length transcripts generated from fruit receptacles^13^. Gene models were predicted according to three lines of evidence, namely, *ab initio* gene prediction, protein-based homology detection, and RNA sequence mapping. Finally, the two results were combined using PASA to obtain the new annotation, which contains 34,007 protein-coding genes and is designated v4.0.a2 (Table S1).

Compared to v4.0.a1, there are 5,419 (18.96%) more genes in v4.0.a2. The locus IDs for the existing genes in v4.0.a1 remain the same in the new version, v4.0.a2, following the format of FvH4_XgXXXXX. The last digit of these gene IDs is always 0. For newly added loci, gene IDs follow the same format, FvH4_XgXXXXX, but have a last digit that is distinct from those of their neighboring genes. For genes whose gene models are modified, the original IDs in v4.0.a1 are retained. For genes that are split into two or more genes, the original ID is assigned to one of the genes, and a new ID is assigned to each of the other genes. For merged genes, one of the original IDs is retained. For removed genes, the IDs are removed and no longer used. To facilitate references to prior *F. vesca* transcriptome datasets, the gene IDs corresponding to the old nomenclature are listed alongside the new gene IDs in Table S2. The statistics comparing v4.0.a1 and v4.0.a2 are shown in Table 1. The average number of exons is increased from 5.5 to 6.6. Approximately three thousand more genes are found to possess 5′ UTRs and/or 3′ UTRs. Alternatively spliced isoforms are also included in this annotation. A total of 64,598 transcripts from 10,176 genes were found, resulting in an average of 1.90 transcripts per locus at the whole-genome scale.

**Table 1:** Summary of the v4.0.a2 annotation

Type	v4.0.a1	v4.0.a2
*Protein-coding genes*		
Number of genes	28,588	34,007
Mean length of genomic loci	3,213	2,953
Mean exon number	5.5	6.6
Mean CDS length	1,178	1,155
Mean length of introns	350	322
Genes with 5′ UTR	16,001	19,711
Genes with 3′ UTR	17,263	20,119
Genes with both 5′ and 3′ UTR	14,657	18,909
Mean 5′ UTR length (bp)	197	225
Mean 3′ UTR length (bp)	336	406
Number of genes with isoforms	—	10,176
Mean isoform number per gene	1.0	1.9
Genes with GO terms	15,208	18,523
Genes with functional annotations	25,095	30,692
Complete BUSCOs	91.1%	98.1%
Fragmented BUSCOs	2.1%	0.8%
Missing BUSCOs	6.8%	1.1%

### Evaluation of annotation v4.0.a2

**Fig. 1:**
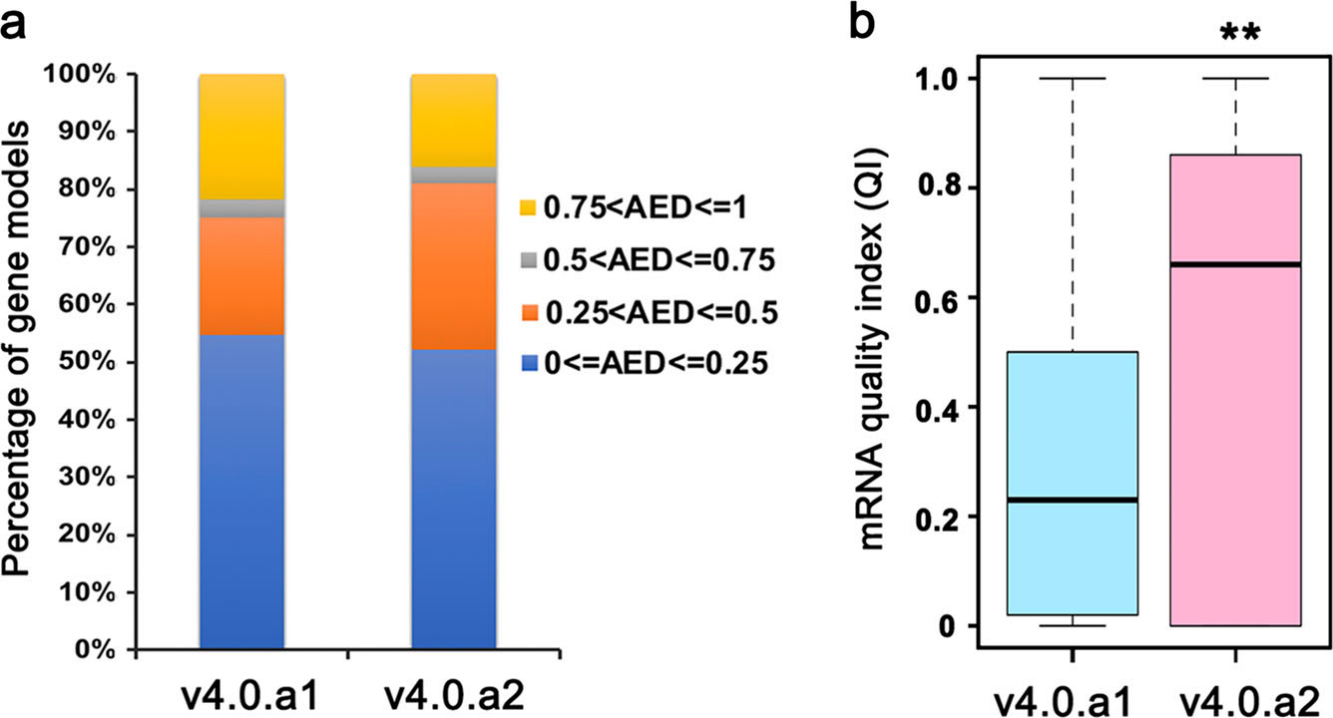
Quality evaluation of v4.0.a1 and v4.0.a2. **a** Bar graph showing the percentages of gene models with different ranges of AED scores in v4.0.a1 and v4.0.a2. For each bar, the four colors from bottom to top indicate genes in the AED ranges 0–0.25, 0.25–0.5, 0.5–0.75, and 0.75–1, respectively. **b** Boxplot showing the mRNA quality index (QI) in v4.0.a1 and v4.0.a2. **, *P* < 0.01, Student’s *t*-test

To evaluate the accuracy of the v4.0.a2 annotation, we used MAKER2 to generate the quality-control metrics for the two annotation versions, including the Annotation Edit Distance (AED) and mRNA quality index (QI)^14^. AED measures the consistency of gene models with evidence alignment. AED scores are between 0 and 1, with an AED of 0 denoting complete agreement with the evidence and 1 indicating complete absence of support for the annotated gene model. QI scores are also between 0 and 1. A higher QI score indicates a greater fraction of exons that match a transcript alignment. Consequently, 80.9% of the v4.0.a2 gene models have AED scores of less than 0.5, whereas 75.1% of the v4.0.a1 gene models have an AED <0.5. In total, 1,711 gene models in v4.0.a2 have AEDs of 0, in contrast to 898 gene models in v4.0.a1 (Fig. 1a). A comparison of the QI score distribution shows that v4.0.a2 contains a significantly greater fraction of gene models supported by the RNA-seq data (Fig. 1b). Furthermore, we used the Benchmarking Universal Single-Copy Orthologs (BUSCO) toolkit^15^ to examine the completeness of each annotation. BUSCO is a measure for the quantitative assessment of genome assembly and annotation completeness based on evolutionarily informed expectations of gene content. The highest BUSCO score is 100%. A higher BUSCO score indicates a better quality of the annotated genome. Of the 1,440 conserved genes, v4.0.a2 harbors 98.1% complete BUSCOs, which is significantly higher than v4.0.a1 (91.1%) (Table 1). These data suggest that v4.0.a2 is greatly improved.

### Prediction of gene functions

To update gene functional annotations of v4.0.a2, all the protein sequences were blasted against the InterPro database using InterProScan^16^. As a result, 30,692 genes contain known protein domains in the Pfam database, compared to 25,095 genes in v4.0.a1 (Table 1). Then, Blast2GO^17^ was used to assign GO categories for all the genes. Consequently, 18,523 genes are allocated to specific GO terms, compared to 15,208 genes in v4.0.a1 (Table 1). Furthermore, transcription factors and protein kinases were also detected and classified by the iTAK pipeline^18^. A total of 1,590 transcription factors and 393 transcriptional regulators were identified in the v4.0.a2 annotation (Table S3). There are 139 more transcription factor genes in v4.0.a2 than v4.0.a1. The size of some gene families changes dramatically. For instance, the members of the family *far-red impaired response 1* (*FAR1*) are increased from 2 to 82. The founding member FAR1 shares similarities to Mutator-like transposases and acts together with *far-red elongated hypocotyls 3* (*FHY3*) in the nucleus to regulate gene expression in the phytochrome A signaling pathway^19,20^. Some other transcription factor families also gain moderately in number, such as the B3 family from 77 to 89, the bHLH family from 102 to 107, and the MYB family from 117 to 121. In addition, there are 1,145 protein kinase encoding genes in v4.0.a2, 92 more than in v4.0.a1 (Table S3). As detailed above, v4.0.a2 contains a large number of genes that are either absent or have inaccurate annotations in v4.0.a1.

### Characterization of the new genes identified in v4.0.a2

**Fig. 2:**
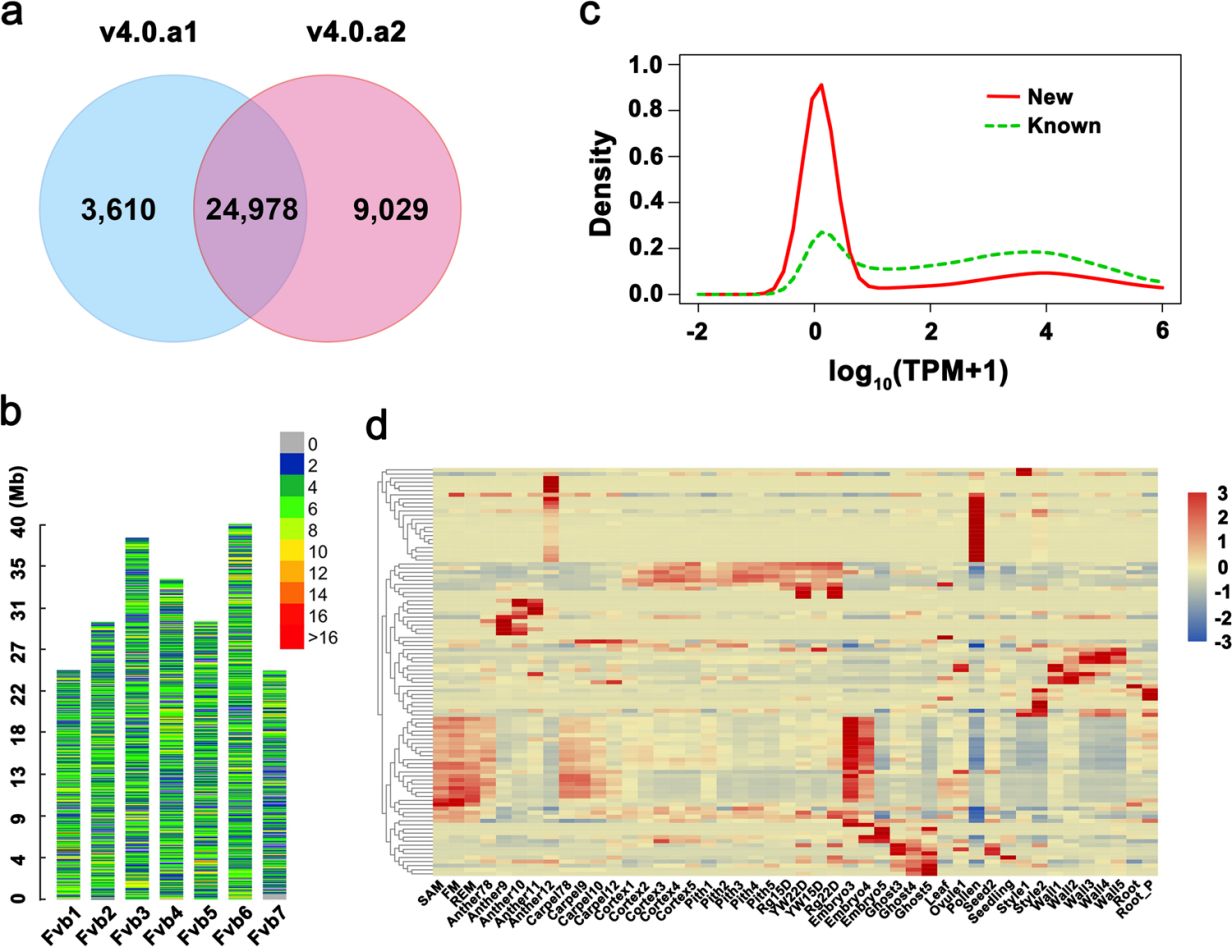
Characterization of the new genes in v4.0.a2. **a** Venn diagram showing the unique and common genes between v4.0.a1 and v4.0.a2. **b** Gene density of the new genes in v4.0.a2 on each chromosome. The number of new genes per 0.1 Mb is shown as the color index. **c** Density plot showing the expression levels of new (red) and known (green) genes in v4.0.a2. The *X*-axis is the log10-transformed TPM+1 for each gene. **d** Heatmap showing the top 100 abundantly expressed genes among the 9,029 new genes in v4.0.a2 across a total of 46 different tissues. The color bar indicates variance-stabilized transformed values on a log2 scale

When comparing the gene models in v4.0.a1 and v4.0.a2 by Cuffcompare, a total of 24,978 genes are shared, accounting for 87.37% of v4.0.a1 and 73.45% of v4.0.a2. In total, 3,610 genes in v4.0.a1 are absent from v4.0.a2; most of them are either not supported by expression or not supported by protein homology evidence. More importantly, v4.0.a2 added 9,029 new genes (Fig. 2b, Table S4); these new genes are from intergenic regions or genic regions with thoroughly different gene models in v4.0.a1. To further characterize these new genes, we examined their distribution across the genome. We found that these genes are evenly distributed in the seven chromosomes (Fig. 2b), ranging from 847 in Chromosome 7 to 1,704 in Chromosome 6. Then, we examined their expression levels in the 46 tissue types according to the RNA-seq data. A total of 4,832 genes (53.52% out of the 9,029 genes) are expressed at a level greater than 1 transcript per million reads (TPM) in at least one of those tissues (Table S4). The distributions of the maximum expression level of these 9,029 genes and the 24,978 shared genes among the tissues were plotted (Fig. 2c). Notably, many new genes are expressed at very low levels or not expressed at all. We hypothesize that these genes should be well supported by the conservation of protein sequences. As expected, 5,793 new genes (64.16%) contain Pfam protein domains^21^, and 2,967 new genes (32.86%) possess GO terms. These genes might be expressed in other unexamined tissues or induced under different growth conditions. Overall, a total of 7,361 new genes (81.53%) are strongly supported by either digital gene expression or conserved protein domains. The expression patterns of the top 100 most abundantly expressed genes in the available transcriptome data were analyzed by hierarchical clustering. As shown in Fig. 2d, a large proportion of them are specifically expressed (Table S4), for instance, in the heart-stage embryo, stage 12 anther, or mature pollen.

### Validation of the selected gene models

**Fig. 3:**
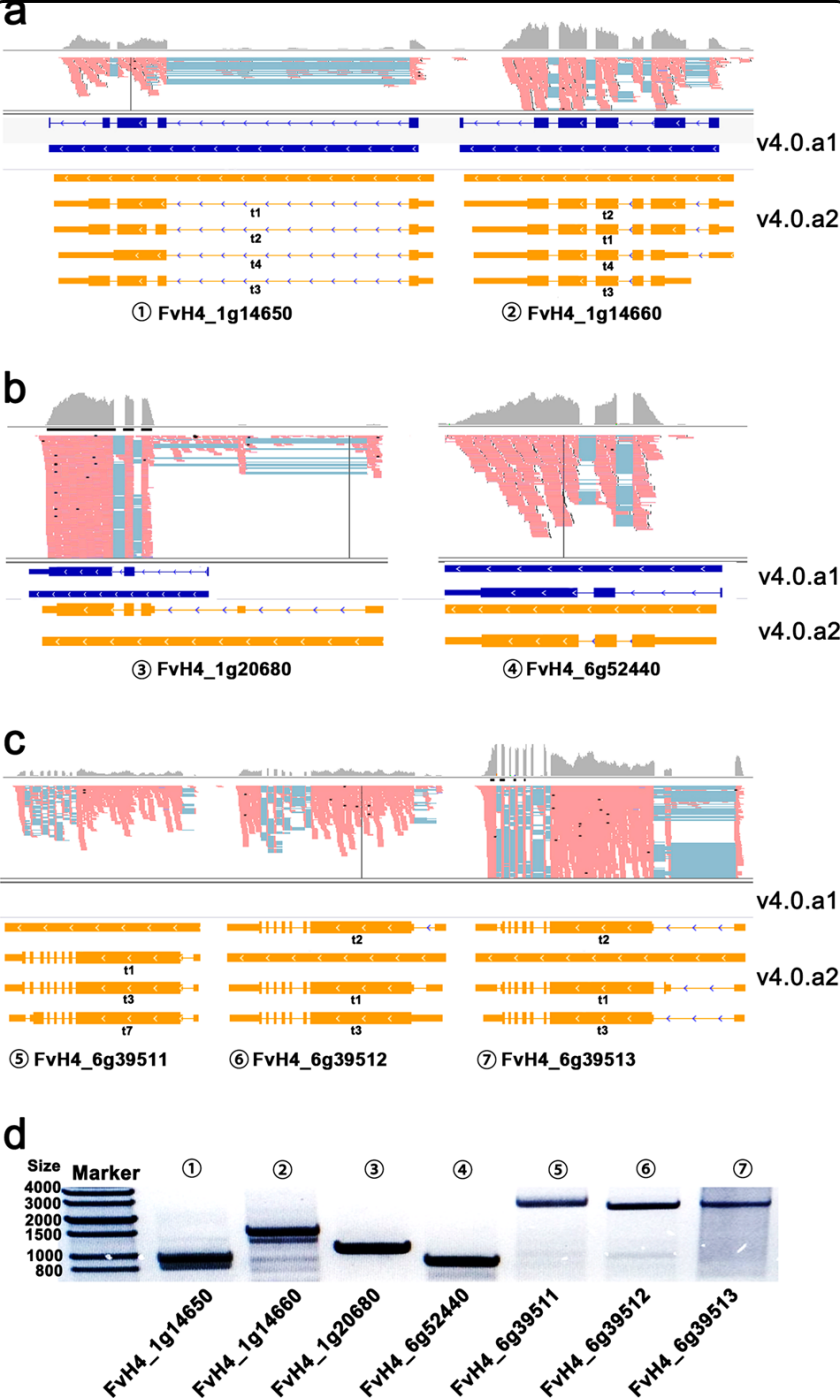
Examples of known genes with improved annotations or newly identified genes. **a** IGV view of the RNA-seq reads for the two adjacent genes (FvH4_1g14650 and FvH4_1g14660) with changed gene models in v4.0.a2 vs v4.0.a1 in the tissue “SAM”. **b** Gene models of two *MYB* genes that were specifically expressed in roots. **c** Gene models of three newly identified genes encoding FAR1 family proteins in the tissue “SAM”. Gray peaks indicate read coverage. Pink bars indicate the aligned reads. Blue bars indicate exons in v4.0.a1. Orange bars indicate exons in v4.0.a2. The thinner orange bars indicate UTRs. “t1”–“t7” under each gene model indicate different isoforms. d, Gel image showing the amplified products of the seven genes. The sizes of the products are 924 bp, 1,473 bp, 1,050 bp, 846 bp, 2,685 bp, 2,505 bp, and 2,601 bp, respectively

To validate the gene models, we randomly inspected seven genes. Some of the gene annotations in v4.0.a1 are not entirely accurate. For example, the transcripts of FvH4_1g14650, the homolog of *AtVRN1* (AT3g18990) needed for repression of *FLC* during vernalization^22^, possess only three or four exons rather than five exons (Fig. 3a). Its adjacent gene FvH4_1g14660, the homolog of *related to vernalization 1* (*RTV1*, AT1G49480) and a nuclear-localized DNA-binding protein^23^, loses its last exon and gains one additional exon after the second exon (Fig. 3a). Two root-specific MYB transcription factor genes are mis-annotated in v4.0.a1 (Fig., 3b); FvH4_1g20680 encodes the homolog of AtMYB93 (AT1G34670), which acts as a negative regulator of lateral root development in *Arabidopsis*^24^, and FvH4_6g52440 encodes the homolog of AtMYB40 (AT5G14340), which is also root-specific in *Arabidopsis*^25^. In addition, some genes with well-conserved domains are absent in v4.0.a1. For example, three adjacent homologous genes of *FAR1* (AT4g15090) are absent in v4.0.a1 (Fig. 3c). These gene models were all validated by PCR amplification (Fig. 3d) and Sanger sequencing using primers shown in Table S5.

### Expression profiles of all the genes according to RNA-seq data

Digital gene expression profiles provide valuable resources for investigating gene functions. Here, we utilized both previously published and newly generated RNA-seq data from 46 tissue types in *F. vesca*, including tissues from flowers and fruit at different developmental stages, seedlings, leaves, meristems, and roots. To facilitate future studies, we created a table showing the expression levels of all the genes across the available tissue types represented by TPM (Table S6). A total of 27,554 genes (81.02% out of the 34,007 genes) are expressed at a level >1 TPM in at least one of these tissues. This table also contains two columns showing short descriptions whenever applicable.

### Annotation of miRNAs and their target genes

**Fig. 4:**
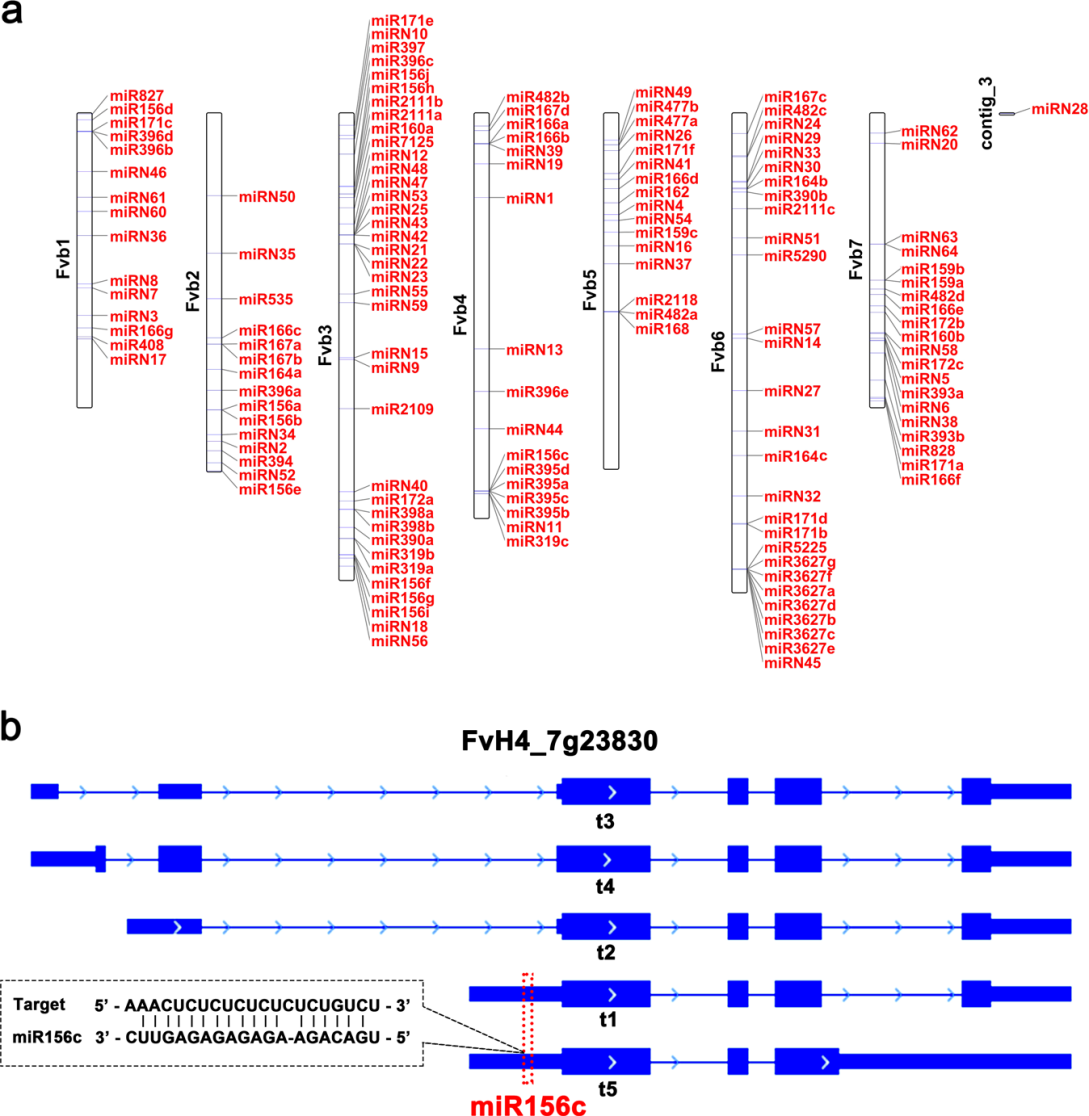
Distribution of the miRNA genes in different chromosomes and one target site of miR156. **a** Distribution of the annotated miRNA genes (both known and new) in different chromosomes in the *F. vesca* V4 genome. **b**, The target site of miR156c in FvH4_7g23830. This gene has five isoforms, two of which are targeted by miR156c at the 5’ UTR. The target sites are indicated by a red dashed box

Previously, miRNAs were globally identified from nine different tissues of *F. vesca* using the initial version of the genome as ref. 26. To locate miRNAs in the V4 genome, nine sRNA libraries from vegetative tissues, flower buds, newly opened flowers and fruit tissues that separated achenes from the receptacle were processed and analyzed following an established protocol^26^. Positions of miRNA genes on strawberry chromosomes (Table S7) were visualized with TBtools^27^ (Fig. 4a). Consequently, we identified 85 conserved miRNA genes encoding 59 unique miRNA sequences belonging to 31 known miRNA families (Fig. 4a, Table S7). A previous study of strawberry miRNAs was based on the first genome of *F. vesca* (v1.0) that was assembled on scaffolds^26^. Compared to the previous results, the same number of known miRNA families (31) is identified, but five new families (miR2118, miR398, miR5290, miR7125, and miR828) are included, while five families (miR1511, miR169, miR399, miR530, and miR845) are lost. Furthermore, we found that some miRNA genes are generated from fewer loci in V4, such as miR156 (from 17 to 10) and miR171 (from 11 to 6). In addition, using the highly stringent criteria established previously^26^, 64 miRNAs are characterized as novel miRNAs and designated fve-miRN1 to miRN64 (Fig. 4a, Table S7). There are more novel miRNA genes in the V4 genome compared to the 33 novel miRNA genes identified previously^26^, perhaps due to the newly augmented ~25 Mb sequences in the V4 genome or the application of new versions of miRNA analysis tools. These conserved and novel miRNA genes are unevenly located in the seven chromosomes; only one miRNA gene (fve-miRN28) is located in the unanchored contig (Fig. 4a).

Finally, the targets of these miRNAs, supported by both software prediction and the degradome sequencing data, are listed (Table S8). In total, 580 target genes are possibly recognized by 30 conserved miRNA families, and 302 genes are the targets of 60 novel miRNAs. Compared to the previous study^26^, 497 more target genes in v4.0.a2 were identified. It was reported that the UTR regions are frequently targeted by miRNAs^28,29^, which may contribute to this remarkable difference, owing to the presence of UTRs in the v4.0.a2 annotation (UTRs were not included in the v1.0 annotation used in the previous *F. vesca* miRNA study^26^). Indeed, some genes gain miRNA target sites in the UTR regions. For instance, FvH4_7g23830, which codes for a nucleotide-sugar transporter, is annotated to have five isoforms, two of which bear target sites of fve-miR156c at the 5′ UTR (Fig. 4b).

## Conclusion

Recently, a high-quality genome of *F*. *vesca*, V4, became available^4^. Here, we generated an improved annotation, called v4.0.a2, for this new genome. We combined two approaches by mapping the previous v2.0.a2 annotation to the V4 genome and reannotating the V4 genome using both Illumina short reads derived from 46 different tissues and PacBio long reads from fruit receptacles. This new annotation, v4.0.a2, is of high quality as validated by MAKER2, GO term assignment, and BUSCO analysis. A total of 9,029 new genes were identified and included in the new annotation. In silico expression patterns of all the genes in the genome are provided. In addition, miRNAs are identified, and their targets are predicted. This new annotation will be a valuable resource for comparative and functional studies in strawberry and its relatives.

## Materials and methods

### Transcriptome datasets used in this study

For the annotation analysis, we collected 97 Illumina-based RNA-seq libraries generated from early-stage fruit, green-stage fruit, turning-stage fruit, different stages of floral organs, floral meristem, receptacle meristem, shoot apical meristem, roots, and a PacBio dataset generated from pooled strawberry fruit at different developmental stages in *F. vesca*^8,9,11–13^. Details of these datasets were described in our previous study^11,30^. In addition, a total of nine small RNA-seq libraries generated from flowers, fruit and vegetative tissues were used for small RNA identification^26^, and three degradome libraries generated from these tissues were used for target validation^26^.

### Pipeline used for reannotation of the *F. vesca* V4 genome

First, the gene models of *F. vesca* annotation version v2.0.a2^30^ were mapped to the V4 genome^4^ using the default FLO pipeline (https://github.com/wurmlab/flo), which is a way of mapping annotations from one assembly to another. In parallel, the previously established annotation pipeline was adopted to predict the gene models using the new genome and the RNA-seq datasets^30^. In brief, the raw reads were filtered using the fastq_quality_filter (-q 28 -p 90) and trimmed using fastx_trimmer built in the FASTX-Toolkit (http://hannonlab.cshl.edu/fastx_toolkit/). Then, the clean reads were aligned to the V4 genome (downloaded from GDR: https://www.rosaceae.org/) using STAR^31^ with the 2-pass mapping mode and following parameters: --alignIntronMin 20, --alignIntronMax 10000. Subsequently, the transcripts were assembled by StringTie^32^ with the default parameters except that the minimum isoform fraction was set to 0.2 (-f 0.2) to remove weakly expressed transcripts. Trinity^33^ was utilized to perform de novo assembly on all RNA-seq reads with default parameters. For the PacBio reads, the RS_IsoSeq pipeline (v2.3) was employed for trimming primers, clustering and polishing SMRT reads, and LoRDEC was utilized to further correct the errors in the PacBio reads by using Illumina reads (parameters: -k 19 -s 3). After correction, the polished PacBio reads were mapped to the V4 genome by GMAP^34^ with >85% alignment coverage and >90% alignment identity. These mapped PacBio reads were used to build high-fidelity gene models by Program to Assemble Spliced Alignments (PASA)^35^ for training of the annotation tools. Finally, the genome-guided and de novo full-length transcripts generated above were collapsed, mapped back to the V4 genome, and reconstructed by PASA to build a comprehensive transcriptome.

Augustus and MAKER2 were employed to generate independent gene models based on the transcriptome evidence. The input data for Augustus were prepared as follows: (1) intron hints generated from mapped RNA-seq reads; (2) intron hints generated from PacBio full-length transcripts by GMAP (http://bioinf.uni-greifswald.de/bioinf/wiki/pmwiki.php?n=Augustus. PacBioGMAP); (3) protein hints generated from mapped UniProt proteins; (4) repeat hints from the RepeatMasker output (http://bioinf.uni-greifswald.de/bioinf/wiki/pmwiki.php?n=Augustus.IncorporateRepeats). The input data for MAKER2 were as follows: (1) trained models from SNAP^36^, GENEMARK^37^ and Augustus with full-length transcripts; (2) the comprehensive transcriptome; (3) UniProt proteins; and (4) the repeat-masked V4 genome.

Next, EVidenceModeler (EVM) was used to combine the v4.0.a1 gene models, MAKER2 gene models, Augustus gene models, mapped gene models from v2.0.a2, and transcripts from Illumina RNA-seq and SMRT with a nonstochastic weighted value into confident consensus gene models. The weight values for each type of evidence were set to 7, 6, 8, 10, 8 and 12, respectively. Finally, PASA was used to improve the EVM gene models by modifying gene structures and adding UTR annotations and alternatively spliced isoforms.

### Identification of miRNAs and their target genes

The identification of strawberry miRNAs followed a workflow that was described previously^38,39^. In brief, the reads generated from the nine tissues^26^ were combined and processed by discarding low-quality reads, adapter trimming, and finally collapsing identical small RNA reads into one using the FASTX-Toolkit (http://hannonlab.cshl.edu/fastx_toolkit/). Then, these collapsed reads were mapped to the V4 genome by Bowtie1^40^ with no mismatches allowed. Next, the sRNAs with a length of 20–22 nucleotides and  ≤20 genomic matches were subjected to screening for stem-loop structures (≤4 mispairings, ≤1 central bulge). Finally, the miRNAs were searched against miRbase (www.mirbase.org, v21) by BLAST to find the conserved miRNAs in plants, allowing up to 2 mismatches. TargetFinder 1.7^41^ and CleaveLand^42^ were used to predict the target genes of the miRNAs among genes in v4.0.a2. Alignment scores up to 5 were used in target prediction^43^; a lower score indicates a better alignment between miRNA and its target.

### Experimental verification of new gene models

Total RNA was isolated from flower buds of YW5AF7, a 7th generation inbred line of the *F. vesca* variety Yellow Wonder, using a Plant Total RNA Isolation Kit (Sangon Biotech, Shanghai, China, No. SK8631). cDNA was synthesized from 1 µg total RNA in a 20-µl solution using a PrimeScript RT reagent kit (TaKaRa, Shiga, Japan, Cat# RR047A). KOD DNA polymerase (TOYOBO Bio-Tech, Cat# F0934K) was used to amplify the coding regions of selected genes for Sanger sequencing with primers listed in Table S5. The sequencing results were aligned with the sequences based on the v4.0.a2 annotation by TBtools^27^.

## Supplementary information

Dataset 1

Dataset 2

Dataset 3

Dataset 4

Dataset 5

Dataset 6

Dataset 7

Dataset 8

## Data Availability

Table S1 is the gff3 file of the new V4 annotation v4.0.a2, which is also freely available through GDR (https://www.rosaceae.org/).
