# Author Correction: Updated annotation of the wild strawberry *Fragaria vesca* V4 genome

**DOI:** 10.1038/s41438-019-0174-y

**Published:** 2019-07-10

**Authors:** Yongping Li, Mengting Pi, Qi Gao, Zhongchi Liu, Chunying Kang

**Affiliations:** 1 Key Laboratory of Horticultural Plant Biology (Ministry of Education), College of Horticulture and Forestry Sciences, Huazhong Agricultural University, Wuhan, Hubei 430070 China; 2 Department of Cell Biology and Molecular Genetics, University of Maryland, College Park, MD 20742 USA

**Keywords:** Transcriptomics, Non-model organisms

**Correction to: Horticulture Research** (2019)6:61

10.1038/s41438-019-0142-6 Published online 01 May 2019

Since the publication of this article, the authors have noticed that the GeneIDs from new and original genome annotations don’t match in Table S6, the correct Table S6 is given here.

The authors would like to apologize for this error.

## Supplementary information

Supplementary Information

